# Complications following locoregional therapy of renal cancer

**DOI:** 10.1186/1470-7330-14-S1-O21

**Published:** 2014-10-09

**Authors:** Miltiadis Krokidis

**Affiliations:** 1 Department of Radiology, Cambridge University Hospitals NHS Foundation Trust, Cambridge, UK

## 

Renal locoregional therapy or local ablation therapy, offers a valid solution for patients with small renal tumours that are not suitable surgical candidates.

Local ablation techniques are usually performed under imaging guidance and are minimal invasive. However, complications may occur even with this minimal invasive treatment.

The most frequent complication of ablation treatment is bleeding. Patients with renal cancer are often on antiplatelet or anticoagulant treatment for other reasons therefore at high risk of bleeding. The formation of a perirenal haematoma is reported in up to 30% from various authors [1-3]. Among the ablation methods cryotherapy appears to be leading to the formation of perirenal bleeding, probably due to the fact that a higher number of probes are usually required. Larger scale bleeding that would require transfusion and embolization is less frequent and is reported in up to 2% in various series [1,4]. Bleeding in such cases may be taking origin form the intercostal arteries or from the ablation site of the renal parenchyma. Bleeding may also be expressed in the form of haematuria in up to 2.5% of the cases particularly when central lesions are treated [5-7]. However, haematuria may be transitional and urine may clear up after 24-48 hours.

Further reported complications may be adjacent organ thermal injury, particularly of the colon, that is reported in up to 1% in the various series [8]. In such cases thermal injury perforation and peritonitis may occur. Hydrodissection with glucose for RFA or CO2 dissection for cryoablation may prevent this complication.

Furthermore, thermal injury of the renal tract may occur and lead to the formation of a urinoma. Cold solution through an antegrade ureteric stent would protect the ureter from thermal injury when central lesions are treated.

Pain post treatment may also occur, however it is usually limited within the first two days. If persistent pain occurs then nerve injury needs to be suspected. Infection in up to 2% has been reported [9] however antibiotics are not routinely administered.

For the treatment of renal cancer surgical resection remains the standard treatment modality. Locoregional therapy has been frequently used to treat renal cancer in patients not suitable for surgical resection. This approach is minimally invasive, however it is not free of complications that need to be recognized and avoided.
